# Long-term atomoxetine-oxybutynin combination use may be beneficial for the prevention of obstructive sleep apnea

**DOI:** 10.1038/s41598-021-91988-5

**Published:** 2021-06-15

**Authors:** Tien-Yu Chen, Chi-Hsiang Chung, Hsin-An Chang, Yu-Chen Kao, Shan-Yueh Chang, Terry B. J. Kuo, Cheryl C. H. Yang, Wu-Chien Chien, Nian-Sheng Tzeng

**Affiliations:** 1 Department of Psychiatry, Tri-Service General Hospital, School of Medicine, National Defense Medical Center, Taipei, Taiwan; 2 Institute of Brain Science, National Yang Ming Chiao Tung University, Taipei 112, Taiwan; 3 Department of Medical Research, Tri-Service General Hospital, National Defense Medical Center, Taipei, Taiwan; 4 School of Public Health, National Defense Medical Center, Taipei, Taiwan; 5 Taiwanese Injury Prevention and Safety Promotion Association, Taipei, Taiwan; 6 Student Counseling Center, National Defense Medical Center, Taipei, Taiwan; 7 Department of Psychiatry, Tri-Service General Hospital, Song-Shan Branch, School of Medicine, National Defense Medical Center, Taipei, Taiwan; 8 Division of Pulmonary and Critical Care Medicine, Department of Internal Medicine, Tri-Service General Hospital, School of Medicine, National Defense Medical Center, Taipei, Taiwan; 9 Graduate Institute of Medical Sciences, National Defense Medical Center, Taipei, Taiwan; 10 Clinical Research Center, Taoyuan Psychiatric Center, Ministry of Health and Welfare, Taoyuan, Taiwan; 11 Graduate Institute of Life Sciences, National Defense Medical Center, Taipei, Taiwan

**Keywords:** Drug discovery, Neuroscience, Medical research, Neurology

## Abstract

One recent study showed that atomoxetine-oxybutynin combination (AOC) use is effective in reducing obstructive sleep apnea (OSA) severity. We used a nationwide database to examine the association between AOC use and the risk of OSA incidence. This retrospective cohort study used Taiwan’s National Health Insurance Research Database between the years 2000 and 2015. The patients who used atomoxetine or oxybutynin were included as an exposed cohort. The exposed and unexposed groups were selected in a ratio of 1:3 with sex, age, and index year matching. We used the multivariate Cox proportional regression model to evaluate the association between AOC use and the risk of an incident diagnosis of OSA. The incidence rates of OSA in the exposed cohort (N = 8940) and the unexposed cohort (N = 26,820), were 21.92 and 22.93 per 100,000 person-years, respectively. The adjusted hazard ratio of oxybutynin use only and AOC with a treatment duration of ≥ 366 days were 0.307 (95% CI 0.204–0.995, *P* = 0.045) and 0.299 (95% CI 0.102–0.933, *P* = 0.002), respectively. Long-term atomoxetine-oxybutynin combination therapy may be beneficial to reduce the risk of obstructive sleep apnea. Further studies to examine these mechanisms are warranted.

## Introduction

Obstructive sleep apnea (OSA) is a common disorder characterized by episodic upper airway obstruction during sleep^1^. The symptoms and signs of OSA include sleep fragmentation, hypoxia, hypercapnia, and increased sympathetic activity during sleep^2^.

Increasing evidence has indicated that OSA is significantly associated with the risk of motor vehicle accidents^3^, cardiovascular diseases^4^, cerebrovascular morbidities^5–7^, and metabolic syndrome^8^. The treatment is considered for patients with apnea–hypopnea index (AHI) ≥ 15 per hour and those with OSA-related symptoms, such as daytime sleepiness and impaired cognitive function^9^.

Several treatment options are available for OSA. Positive airway pressure (PAP) therapy is efficacious and can improve both subjective and objective sleepiness and daytime functions^10^. However, poor adherence to long-term PAP therapy is a limitation^11^. The oral appliance therapy could be used for mild-to-moderate OSA patients who are unable to use PAP; however, long-term use of the appliance may cause dental side effects^12^. Surgical interventions like uvulopharyngopalatoplasty and hypoglossal nerve stimulation may be helpful for patients with favorable anatomy^13,14^.

The evidence of pharmacologic intervention for the treatment of OSA is still sparse. A systematic review found some drugs like acetazolamide, tramazoline, liraglutide, spironolactone/furosemide, dronabinol, zonisamide, phentermine, spironolactone, and ondansetron/fluoxetine may have benefits on reducing AHI compared to placebo. However, most of the selected trials were not adequately evaluated^15^.

One recent well-designed trial has shown that the use of atomoxetine 80 mg and oxybutynin 5 mg, atomoxetine-oxybutynin combination (AOC) treatment, but not monotherapy, appear to be immediately effective in lowering the AHI in patients with OSA by increasing the genioglossus responsiveness^16^. To examine this, we used a nationwide population-based registry dataset to evaluate the relationship between the use of atomoxetine/oxybutynin and the risk of incident diagnosis of OSA.

## Results

### Sample characteristics

Table 1 shows the baseline characteristics of sex, age, comorbidities, urbanization, area of residence, monthly insured premiums of the patients in the exposed and unexposed cohort. When compared to the unexposed cohort, the participants in the exposed cohort tended to have higher rates of attention-deficit hyperactivity disorder (ADHD), overactive bladder syndrome (OAB), diabetes mellitus (DM), hypertension, and Charlson Comorbidity Index, revised (CCI-R) scored 0 and 3, and lower rates of hyperlipidemia and coronary artery disease (CAD). The participants in the exposed cohort tended to have slightly higher rates of the monthly insured premiums of the NT$ ≥ 35,000, and 18,000–34,999. The participants in the exposed cohort tended to have higher rates of living in the north and east of Taiwan, in urbanization level 1 and 2 areas, and searching medical help from the medical centers and regional hospitals than the controls.

**Table 1:** Characteristics of study at the baseline.

Variables	Exposed cohort*		Unexposed cohort		*P*
	n	%	n	%	
Total	8940	25.00	26,820	75.00	
**Sex**					0.999
Male	5068	56.69	15,204	56.69	
Female	3872	43.31	11,616	43.31	
Age (years)	62.36 ± 18.55		60.97 ± 18.72		0.149
**Age group (years)**					0.999
0–12	244	2.73	732	2.73	
13–17	120	1.34	360	1.34	
18–44	1093	12.23	3279	12.23	
45–64	2349	26.28	7047	26.28	
≥ 65	5134	57.43	15,402	57.43	
**Marital status**					0.540
Without	4875	54.53	14,725	54.90	
With	4065	45.47	12,095	45.10	
**Educational years**					0.699
< 12	4972	55.62	14,979	55.85	
≥ 12	3968	44.38	11,841	44.15	
**Insured premium (NT$)**					0.001
< 18,000	8808	98.52	26,548	98.99	
18,000–34,999	115	1.29	224	0.84	
≥ 35,000	17	0.19	48	0.18	
ADHD	851	9.52	277	1.03	< 0.001
OAB	890	9.96	270	1.01	< 0.001
DM	1826	20.43	3740	13.94	< 0.001
Hyperlipidemia	112	1.25	741	2.76	< 0.001
Hypertension	1812	20.27	4796	17.88	< 0.001
CAD	526	5.88	2374	8.85	< 0.001
**CCI_R**					< 0.001
0	5905	66.05	16,766	62.51	
1	1252	14.00	5259	19.61	
2	586	6.55	1831	6.83	
3	786	8.79	1652	6.16	
≥ 4	411	4.60	1312	4.89	
**Location**					< 0.001
Northern Taiwan	3788	42.37	10,533	39.27	
Middle Taiwan	2317	25.92	7400	27.59	
Southern Taiwan	2197	24.57	7037	26.24	
Eastern Taiwan	612	6.85	1699	6.33	
Outer islands	26	0.29	151	0.56	
**Urbanization level**					< 0.001
1 (The highest)	3192	35.70	8901	33.19	
2	3864	43.22	11,492	42.85	
3	599	6.70	1894	7.06	
4 (the lowest)	1285	14.37	4533	16.90	
**Level of medical care**					< 0.001
Medical center	3667	41.02	8457	31.53	
Regional hospital	3928	43.94	8378	31.24	
Local hospital	1345	15.04	9985	37.23	

*P:* Chi-square/Fisher exact test on categorical variables and t-test on continue variables; ADHD: attention deficit hyperactivity disorder; OAB: overactive bladder; DM: diabetes mellitus; CAD: coronary artery disease; CCI_R: Charlson Comorbidity Index, revised.

*Exposed cohort: subjects exposed to atomoxetine or oxybutynin during the study period.

### Kaplan–Meier model for the cumulative risk of OSA

There was no significant difference between the cumulative incident diagnosis of OSA in the participants in exposed and unexposed cohorts (*P*-value of the log-rank test = 0.336).

### Factors contributing to OSA

Table 2 depicts that the Cox regression analysis of the factors contributing to OSA. The crude hazard ratio (HR) for patients with atomoxetine or oxybutynin use and the incident diagnosis of OSA was 0.896 (95% CI 0.681–1.323, *P* = 0.242). After adjusting for gender, age, marital status, educational years, monthly income, ADHD, OAB, DM, hyperlipidemia, hypertension, CAD, CCI_R scores, urbanization level of residence, and level of medical care, the adjusted HR (aHR) was 0.843 (95% CI 0.517–1.934, *P* = 0.243). Furthermore, male gender, and patients in medical center were associated with a higher risk of incident diagnosis of OSA; the aHR were 1.86 (95% CI 1.223–2.828, *P* = 0.004) and 3.007 (95% CI 1.509–6.277, *P* = 0.002), respectively. In addition, patients aged ≥ 65 and CCI_R score of 1 were associated with a lower risk of OSA with the aHR as 0.208 (95% CI 0.062–0.696; *P* = 0.011) and 0.692 (95% CI 0.297–0.996; *P* = 0.044), respectively.

**Table 2:** Cox regression analysis of factors contributing to obstructive sleep apnea.

Variables	Crude HR	95% CI		*P*	aHR	95% CI		*P*
**Atomoxetine/oxybutynin**								
No use	Reference				Reference			
Either monotherapy or AOC	0.896	0.681	1.323	0.242	0.846	0.517	1.934	0.243
**Gender**								
Male	1.771	1.166	2.688	0.007	1.860	1.223	2.828	**0.004**
Female	Reference				Reference			
**Age (years)**								
0–12	Reference				Reference			
13–17	0.000	–	–	0.947	0.000	–	–	0.963
18–44	0.479	0.141	1.627	0.238	0.649	0.191	2.213	0.490
45–64	0.286	0.088	0.956	0.042	0.421	0.124	1.428	0.165
≥ 65	0.183	0.041	0.432	0.001	0.208	0.062	0.696	**0.011**
**Marital status**								
Without	1.382	0.811	1.970	0.787	1.562	0.852	2.030	0.897
**Educational years**								
≥ 12	1.301	0.705	1.744	0.652	1.453	0.776	1.986	0.742
**Insured premium (NT$)**								
< 18,000	Reference				Reference			
18,000–34,999	0.712	0.099	5.105	0.735	0.681	0.095	4.888	0.702
≥ 35,000	0.000	–	–	0.963	0.000	–	–	0.977
ADHD	0.000	–	–	0.997	0.000	–	–	0.993
OAB	0.000	–	–	0.997	0.000	–	–	0.995
DM	0.797	0.479	1.326	0.383	0.946	0.558	1.604	0.837
Hyperlipidemia	1.240	0.456	3.374	0.673	1.390	0.500	3.865	0.527
Hypertension	0.793	0.507	1.239	0.308	0.891	0.555	1.430	0.631
CAD	0.172	0.042	0.698	0.014	0.160	0.039	1.057	0.061
**CCI_R**								
0	Reference				Reference			
1	0.946	0.743	1.176	0.092	0.692	0.297	0.996	**0.044**
2	1.087	0.304	1.453	0.306	1.097	0.345	1.533	0.265
3	1.369	0.116	1.765	0.533	1.264	0.567	2.005	0.397
≥ 4	1.664	0.012	3.930	0.211	1.857	0.735	3.798	0.184
**Urbanization level**								
1 (the highest)	2.574	1.287	5.147	0.008	1.522	0.707	3.277	0.283
2	1.847	0.935	3.651	0.077	1.326	0.645	2.730	0.443
3	1.232	0.421	3.606	0.703	1.099	0.375	3.221	0.863
4 (the lowest)	Reference				Reference			
**Level of medical care**								
Hospital center	3.361	1.753	6.442	< 0.001	3.007	1.509	6.277	**0.002**
Regional hospital	1.765	0.906	3.442	0.095	1.680	0.852	3.312	0.134
Local hospital	Reference				Reference			

Location not included given collinearity with urbanization level.

aHR: adjusted Hazard ratio (adjusted for the variables listed in Table 1), AOC: atomoxetine-oxybutynin combination; CI: confidence interval; ADHD: attention deficit hyperactivity disorder; OAB: overactive bladder; DM: diabetes mellitus; CAD: coronary artery disease; CCI_R: Charlson Comorbidity Index, revised.

### Effects of atomoxetine/oxybutynin use on the risk of obstructive sleep apnea

Table 3 presents 4 models of different ways of medication use. The incidence rates of OSA diagnosis in the exposed (either one or combination use of atomoxetine and oxybutynin) (N = 8940) and the unexposed cohort (N = 26,820), were 21.92 and 22.93 per 100,000 person-years, with the aHR as 0.846 (95% CI 0.517–1.934; *P* = 0.243) when unexposed participants were taken as reference. The aHR of either one or AOC with the treatment duration of ≥ 366 days was 0.401 (95% CI 0.222–0.997; *P* = 0.049). Furthermore, the aHR of oxybutynin use only and AOC with a treatment duration of ≥ 366 days were 0.307 (95% CI 0.204–0.995, *P* = 0.045) and 0.299 (95% CI 0.102–0.933, *P* = 0.002), respectively.

**Table 3:** Effects of atomoxetine/oxybutynin use on the risk of obstructive sleep apnea.

Model	Atomoxetine/oxybutynin use	Population	Events	PYs	Rate (per 10^5^ PYs)	aHR	95% CI		*P*
1	No use	26,820	78	340,161.37	22.93	Reference			
	Either monotherapy or AOC	8940	25	114,053.98	21.92	0.846	0.517	1.934	0.243
2	No use	26,820	78	340,161.37	22.93	Reference			
	Atomoxetine only	4301	13	48,121.81	27.01	1.264	0.862	2.894	0.392
	Oxybutynin only	4502	10	53,454.01	18.71	0.782	0.503	1.880	0.241
	AOC	137	2	12,478.16	16.03	0.466	0.264	1.307	0.181
3	No use	26,820	78	340,161.37	22.93	Reference			
	Either monotherapy or AOC, 30–365 days	2,75	16	47,505.07	33.68	1.463	0.672	2.565	0.711
	Either monotherapy or AOC, ≥ 366 days	5965	9	66,548.91	13.52	0.401	0.222	0.997	**0.049**
4	No use	26,820	78	340,161.37	22.93	Reference			
	Atomoxetine only, 30–365 days	1312	9	21,101.78	42.65	2.067	0.975	4.121	0.896
	Atomoxetine only, ≥ 366 days	2989	4	27,020.03	14.80	0.438	0.330	1.996	0.245
	Oxybutynin only, 30–365 days	1583	6	21,981.04	27.30	1.385	0.904	2.670	0.332
	Oxybutynin only, ≥ 366 days	2919	4	31,472.97	12.71	0.307	0.204	0.995	**0.045**
	AOC, 30–365 days	80	1	4422.25	22.61	1.154	0.892	1.501	0.197
	AOC, ≥ 366 days	57	1	8055.91	12.41	0.299	0.102	0.933	**0.002**

PYs: person-years; aHR: adjusted Hazard ratio (adjusted for the variables listed in Table 1), CI: confidence interval; AOC: atomoxetine-oxybutynin combination.

## Discussion

We used a population-based, retrospective cohort study designed with a large sample size and long-term follow-up duration to demonstrate that patients using either oxybutynin or AOC for more than 365 days were associated with a decreased risk of an incident diagnosis of OSA.

In this study, we have examined the association of the use of atomoxetine and oxybutynin with the risk of an incident diagnosis of OSA. After adjusting covariates, the aHR was 0.846 (95% CI 0.517–1.934, *P* = 0.243) in the exposed cohort, when compared with the unexposed cohort. In other words, no significant difference was observed in the risk of an incident diagnosis of OSA between these two groups. However, patients with either oxybutynin or AOC use for more than 365 days had decreased risk of incident diagnosis of OSA, with aHR of 0.307 (95% CI 0.102–0.933, *P* = 0.002) and 0.299 (95% CI 0.204–0.905, *P* = 0.002), respectively.

The findings of this study found that the use of AOC may reduce the incidence of OSA based on a very preliminary clinical work, which suggests that one-night use of AOC resulted in a ≥ 50% reduction of AHI in OSA patients, but neither atomoxetine nor oxybutynin reduced the AHI when administered alone^16^. In comparison to the previous trial^16^, this study was a retrospective cohort study involving a large nationwide database to present real-world data; the previous study was a pilot clinical trial with a small sample size. In addition, we found a protective effect in terms of reducing the risk of an incident diagnosis of OSA with long-term AOC use (≥ 366 days). The results indicated that AOC use for ≥ 365 days might benefit upper airway function during sleep and reduce the risk of OSA. Finally, our study found that long-term use (≥ 366 days) of oxybutynin, but not atomoxetine, had borderline significance (*P* = 0.045) in reducing the risk of an incident diagnosis of OSA. Although the previous clinical study did not find significant benefits with the use of oxybutynin alone^16^, our study provides some insight into the role of oxybutynin in OSA.

In addition, Table 2 shows that patients aged ≥ 65 were associated with a lower risk of OSA. However, some studies have demonstrated that the prevalence of OSA is higher in elderly patients^17,18^. There are several reasons for this phenomenon. First, some of our patients were prescribed atomoxetine or oxybutynin, and the use of such medications may influence the occurrence of OSA. Second, the home sleep test was not included in the National Health Insurance (NHI) in Taiwan. Besides, it is not convenient for the elderly to perform overnight polysomnography in hospitals. These reasons might lead to a lower OSA diagnostic rate in the elderly in Taiwan.

Furthermore, Table 3 shows that the prevalence of OSA in the exposed and unexposed cohorts was 0.28% and 0.29%, respectively. The prevalence of OSA in our study was lower than that reported in other studies^19,20^. This finding can be explained by the following reasons. First, the home sleep test is not popular in Taiwan, and it decreases the willingness for patients to undergo OSA evaluation. Second, we used strict criteria for OSA enrollment in our study, in which each enrolled patient with a diagnosis of OSA was required to have a polysomnography examination record from the database within one year before or after the OSA occurred during the study period. This enrollment strategy might have contributed to the lower prevalence of OSA in this study. Moreover, a low prevalence rate and underdiagnosed OSA have also been reported in other Asian countries^21,22^. It is necessary to highlight the importance of OSA in the general population and among clinicians in Asia.

### Possible mechanisms for the decreased risk of OSA in response to long-term AOC use

The mechanisms underlying the association between the long-term use of oxybutynin and AOC and the reduced risk of an incident diagnosis of OSA are still unclear. However, several possible mechanisms may explain the results. The research group that conducted a previous pilot study on AOC use and the change in OSA severity further investigated the possible effects of atomoxetine and oxybutynin on the respiratory tract^23^. Atomoxetine is a selective norepinephrine reuptake inhibitor that is used in patients with attention-deficit hyperactivity disorder. Its effect of increasing the level of norepinephrine during sleep could stimulate the motoneurons of the upper airway and reduce airway collapsibility^24–26^. In a recent study, atomoxetine alone significantly reduced the arousal threshold and stability of ventilatory control and improved collapsibility, but not muscle compensation during spontaneous breathing while sleeping^23^. Oxybutynin is an antimuscarinic agent frequently used for overactive bladder. The antimuscarinic receptor M2 is one of the few receptors expressed in both the premotor and motor areas of the hypoglossal motor nucleus, and it influences the genioglossus muscle of the tongue^27,28^. In a recent study, compared with placebo, oxybutynin alone improved collapsibility but not muscle compensation during spontaneous breathing while sleeping^23^. The concurrent use of atomoxetine and oxybutynin might enhance upper airway muscle activity and reduce the incidence of OSA.

We should also mention the possible side effects of atomoxetine and oxybutynin if these medications are considered for a long-term use. In general, atomoxetine is relatively safe for patients with ADHD. However, its safety in patients without ADHD is not clear, and the use of atomoxetine may increase blood pressure and heart rate^29^. Oxybutynin is an antimuscarinic agent that might cause significant cognitive deterioration even in short-term use. Although AOC may have a certain value for the development of OSA, the side effects of these medications should be closely monitored.

### Limitations

This study has several limitations. First, the diagnosis of OSA was obtained based on the ICD-9-CM codes of OSA and record of doing a polysomnography exam within one year before or after OSA diagnostic code instead of the polysomnography reports. These data may not be accurate enough for outcome measurement. Furthermore, the severity of OSA and data of body mass index could not be examined in our study. However, the National Health Insurance Administration in Taiwan has made every effort to verify the exactness of the diagnoses in the database. Additionally, to further ensure accuracy, we used the previously published method with an accuracy rate of 87% to define the OSA cases^30^. Second, the use of atomoxetine and oxybutynin was assessed based on prescription records, but the actual dosage used, time of use, and rate of refills were unknown. However, we compared the different durations of medication prescription to minimize the impact of this limitation. Third, the reasons for taking atomoxetine-oxybutynin were not for OSA. Thus, the cases and controls differ in some ways that may bias the results which cannot be fully accounted for in the analyses. However, we included several important covariates like DM, CAD, hypertension to minimize the impacts. Fourth, several important unmeasured confounding factors that may have affected the results. For example, body mass index, smoking habit, and alcohol consumption are related to the incidence of OSA and those factors cannot be assessed by the National Health Insurance Research Database (NHIRD). Since the use of atomoxetine and oxybutynin for treating OSA is a novel and important issue, the results of this nationwide population-based, retrospective cohort study is still important. Finally, the actual mechanisms related to the association between the use of atomoxetine and oxybutynin and OSA risk are still not clear. Though we provided some possible mechanisms in the discussion, further investigation is warranted to shed more light on this issue.

In conclusion, this study provides evidence that long-term (≥ 366 days) atomoxetine-oxybutynin combination use may not increase the risk of OSA, but may be beneficial for its prevention.

## Methods

### Data sources

The NHI program was launched in Taiwan in 1995, and as of June 2009, including contracts with 97% of the medical providers with approximately 23 million beneficiaries, or more than 99% of the entire population^31,32^. The NHIRD, which contains all the claims data of the beneficiaries, uses the International Classification of Diseases, 9th Revision, Clinical Modification (ICD-9-CM) codes to record the diagnoses^33^. The details of the program have been documented in previous studies^34–43^.

A subset of NHIRD, the Longitudinal Health Insurance Database (LHID) of two million randomized, sampled individuals between the years 2000 and 2015, was used to study the association between atomoxetine or oxybutynin and the risk of an incident diagnosis of OSA. The present study used the NHIRD to identify patients with the use of atomoxetine and oxybutynin.

### Ethical statement

This study was approved by the Institutional Review Board of the Tri-Service General Hospital and waived the need for written informed consent (IRB No. B-109-21). All research methods were carried out following the relevant guidelines and regulations.

### Study design and sampled participants

This study used a population-based, matched-cohort design. Patients who had used atomoxetine or oxybutynin for at least 30 days were enrolled from the LHID between January 1, 2000 and December 31, 2015. The exclusion criteria were as follows: (1) participants who had used atomoxetine or oxybutynin before the cohort entry date, (2) participants who had used atomoxetine or oxybutynin for less than 30 days, (3) patients diagnosed with OSA before the cohort entry date, (4) patients diagnosed with OSA before the prescription of atomoxetine or oxybutynin, (5) patients diagnosed with narcolepsy, ICD-9-CM code 347, and (6) missing data. The cohort entry date was defined as the first prescription date of atomoxetine or oxybutynin during the study period. The exposed and unexposed to atomoxetine or oxybutynin groups were selected in a ratio of 1:3 with sex, age, and index year matching. They were followed until the development of OSA, withdrawal from the NHI program, or till the end of the study.

### Major outcomes

The study outcome was the incident diagnosis of OSA, which was defined as patients diagnosed with one of the following ICD-9-CM codes: 327.23, 780.51, 780.53, and 780.57. Each enrolled case with a diagnosis of OSA was required to have a polysomnography exam record from the database within one year before or after OSA occurred during the study period, regarding one previous study^44^. The accuracy of the diagnosis of OSA with the above enrollment has been validated as 87%^30^. The date of the first prescription of atomoxetine or oxybutynin was defined as the index date for the patient.

### Covariates

The covariates included sex, age groups (0–12, 13–17, 18–44, 45–64, ≥ 65 years), marital status, educational years (< 12 years and ≥ 12 years), monthly income (in New Taiwan Dollars [NT$]; < 18,000, 18,000–34,999, ≥ 35,000), geographical area of residence (north, center, south, and east of Taiwan), urbanization level of residence (levels 1 to 4), and level of medical care. The urbanization level of residence was defined according to the population and various indicators of the level of development. Level 1 was defined as a population of > 1,250,000, and a specific designation as political, economic, cultural, and metropolitan development. Level 2 was defined as a population between 500,000 and 1,249,999, and as playing an important role in politics, economy, and culture. Urbanization levels 3 and 4 were defined as a population between 149,999 and 499,999, and < 149,999, respectively. The covariates were referenced with previous articles related to studies of sleep-related topics by using the NHIRD^42,45^.

### Comorbidity

**Figure 1:**
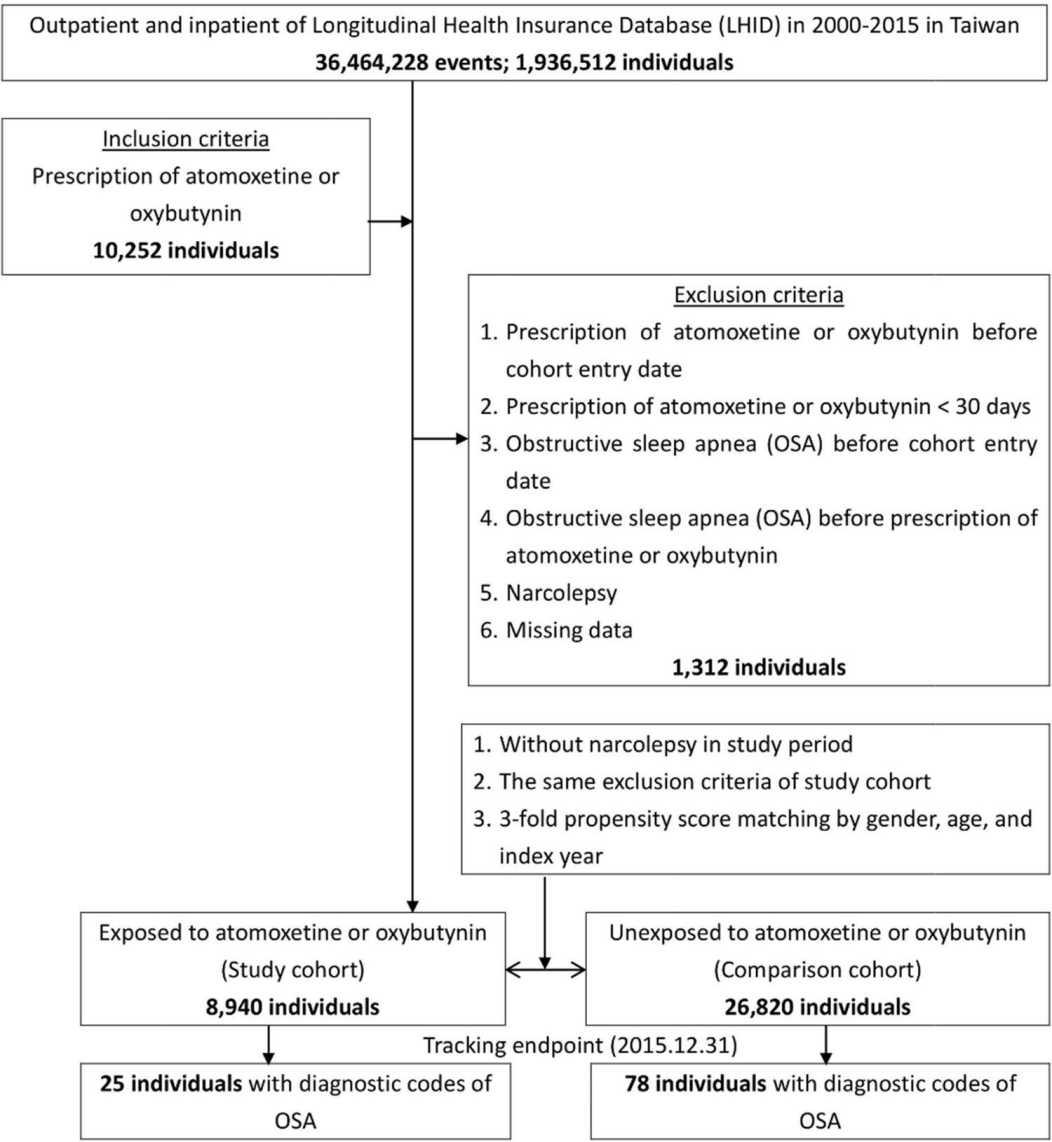
The flowchart of the study sample selection and study design.

We included the following comorbidities in the analysis: ADHD (ICD-9-CM: 314), OAB (ICD-9-CM: 596.51), DM (ICD-9-CM:250), hyperlipidemia (ICD-9-CM:272.x), hypertension (ICD-9-CM:401.1, 401.9, 402.10, 402.90, 404.10, 404.90, 405.1, 405.9), and CAD (ICD-9-CM: 411, 413, 414). Moreover, we also used the Charlson Comorbidity Index (CCI, scored 0, 1, 2, 3, ≥ 4), which categorizes the comorbidities using the ICD-9-CM codes, scores each comorbidity category, and combines all the scores to calculate a single comorbidity score. A score of zero indicates that no comorbidities were found, and higher scores indicate higher comorbidity burdens^46–48^. We removed the items of DM, hypertension, and CAD from the items of CCI, and renamed it as CCI_R, to prevent the multicollinearity with other comorbidities. Figure 1 presents a detailed flowchart regarding participant selection and the study design.

### Statistical analysis

All statistical analyses were performed using SPSS for Windows, version 22.0 (IBM Corp., Armonk, NY). We used χ^2^ and t-tests to evaluate the distributions of the categorical and continuous variables, respectively, with the Fischer exact examination. The multivariate Cox proportional hazards regression analysis was used to determine the risk of an incident diagnosis of OSA, and the results are presented using hazard ratio (HR) with 95% confidence interval (CI). We adjusted for the following potential confounders: sex, age, marital status, educational years, monthly income, ADHD, OAB, DM, hyperlipidemia, hypertension, CAD, CCI_R scores, urbanization level of the residence, and level of care. According to the multicollinearity between the geographical area and urbanization level of the residence, we chose the urbanization level of the residence for adjustment. The difference in the risk of an incident diagnosis of OSA between the exposed cohort and unexposed cohort was estimated using the Kaplan–Meier method with the log-rank test. We further categorized drug use as short-term (30–365 days) and long-term (≥ 366 days) use before the index date. Four different models were used for different patterns of atomoxetine/oxybutynin use in the determination of the association with an incident diagnosis of OSA. Model 1 investigated the difference between no use and either monotherapy or AOC. Model 2 further divided the groups into no use, atomoxetine use only, oxybutynin use only, and the use of AOC. Model 3 incorporated the duration of medication use and divided the groups into no use, either monotherapy or AOC for 30–365 days, and ≥ 366 days. Model 4 investigated the duration of each medication used with the groups as no use, atomoxetine use only for 30–365 days, and ≥ 366 days, oxybutynin use only for 30–365 days and ≥ 366 days, and AOC therapy for 30–365 days and ≥ 366 days. A 2-tailed *p*-value of < 0.05 indicated statistical significance.
